# Proteolytic and non-proteolytic mechanisms of keratin degradation in *Onygena corvina* revealed by a proteogenomic approach

**DOI:** 10.1128/aem.01727-25

**Published:** 2025-11-28

**Authors:** Siddhi Pavale, Clémentine Isembart, Volha Shapaval, Tina R. Tuveng, Sabina Leanti La Rosa, Vincent G. H. Eijsink

**Affiliations:** 1 Faculty of Chemistry, Biotechnology and Food Science, Norwegian University of Life Sciences (NMBU), Ås, Norway; 2 Faculty of Science and Technology, Norwegian University of Life Sciences (NMBU), Ås, Norway; Universidad de los Andes, Bogotá, Colombia

**Keywords:** proteomics, wool meal, feather meal, keratin degradation, *Onygena corvina*

## Abstract

Keratin-rich by-products from the poultry, textile, and leather industries pose a significant challenge for sustainable waste management due to their highly recalcitrant nature. While microbial degradation of keratin has been studied and may offer a viable solution, the complex enzyme machineries that are potentially needed to degrade these recalcitrant by-products remain partly unknown. In this study, we employed a high-resolution proteogenomic approach to characterize the keratinolytic machinery of *Onygena corvina*, a non-pathogenic saprophytic fungus. Using a membrane agar plate method with insoluble substrates, we obtained secretomes enriched in secreted and substrate-bound proteins during growth on α- and β-keratin-rich substrates, specifically wool and feather meal. Our findings reveal that *O. corvina* has a richer proteolytic machinery than previously reported, including enzymes that are used across keratin types, as well as enzymes that are specifically targeted to either α- or β-keratin. In addition to proteases, the secretomes contain numerous other proteins, including cell wall-modifying enzymes, oxidoreductases, esterases, phosphatases, and sialidases that are involved in the deconstruction of keratin. We propose that these additional enzymes destabilize keratin through a combination of mechanical keratinolysis, removal of post-translational modifications, reduction of disulfide bonds, and cleavage of isopeptide bonds, thereby enhancing proteolytic accessibility. Interestingly, keratin degradation by *O. corvina* was most efficient when using mixed substrates containing both feather and wool meal. These novel insights into the keratinolytic system of *O. corvina* underscore the importance of considering synergistic enzyme interactions when developing biotechnological approaches for the valorization of keratin-rich byproducts.

**IMPORTANCE:**

Keratin-rich by-products from agro-industrial processes are generated in large volumes and present a significant environmental burden due to their recalcitrant nature. Microbial degradation offers a promising solution, but the mechanisms involved in keratin decomposition remain elusive. In this study, we show that the saprophytic fungus *O. corvina* secretes a diverse and specialized enzymatic arsenal when grown on keratin-rich substrates, such as feather (β-keratin) and wool (α-keratin) meal. Its secretome includes both shared and keratin-type specific proteases, along with accessory proteins, such as oxidoreductases, esterases, phosphatases, and sialidases, that aid in substrate destabilization. Our findings uncover the complex enzymatic system driving keratinolysis in this fungus and provide a foundation for developing sustainable, enzyme-based strategies to valorize keratin-rich waste.

## INTRODUCTION

Keratin is a fibrous structural protein that is predominantly found in animal-derived hard tissues, such as feathers, wool, hair, horns, and hooves (1). Annually, over 30 billion tons of keratin-rich byproducts are generated worldwide, primarily from the poultry, textile, and leather industries (2, 3). Among these, feathers and wool constitute the major sources of keratin-rich biomass, offering significant potential for the conversion into biodegradable materials, biofertilizers, animal feed, and bioactive peptides (4). Despite this, efficient valorization of keratinous byproducts remains a challenge due to the highly recalcitrant structure of keratin, which limits its degradation by conventional proteases. Keratin is composed of intermediate filaments that are bundled into fibrous structures, and its structure is stabilized by disulfide bonds, hydrogen bonding, and hydrophobic interactions. α-keratin, which is rich in α-helices and the predominant form in wool and hair, exhibits greater resistance to enzymatic degradation than β-keratin, which is rich in β-strands and the primary constituent of feathers (5–7).

Conventional keratin-rich byproduct processing strategies include thermal, chemical, and mechanical treatments, which are energy-intensive and environmentally detrimental, while yielding products of limited value (4). As a sustainable and economically viable alternative, microbial degradation of keratin has emerged as a promising approach. Various microorganisms, including bacteria, fungi, and actinomycetes, have demonstrated keratinolytic capabilities (8). The first report of microbial keratin degradation dates back to the late 19th century with the discovery of keratinolytic activity in a saprophytic fungus, *Onygena equina* (9, 10). Since then, extensive studies have sought to identify and characterize keratin-degrading enzymes, particularly proteases. It has been suggested that degradation of keratin requires a minimum of three distinct types of proteases, including *endo*-acting, *exo*-acting, and oligopeptide-acting enzymes (11–13). However, proteases alone are insufficient to efficiently degrade native keratin due to its extensively cross-linked structure, which limits enzyme accessibility. Indeed, effective keratin degradation requires the cooperative action of multiple enzymes, including disulfide bond-reducing enzymes, such as disulfide reductases, that loosen keratin’s cross-linked structure (14–16). Studies of dermatophytes, such as *Trichophyton rubrum,* have shown that these fungi combine invasive growth through specialized hyphal structures with a multi-enzyme strategy involving a diverse repertoire of secreted proteases, oxidoreductases, and other hydrolases to infect keratinized structures like the epidermal stratum corneum, hair, and nails (17, 18).

Despite these insights, the development of cell-free enzyme preparations for keratin valorization has largely focused on proteases, overlooking the broader enzyme repertoire that may be needed to achieve efficient depolymerization (19). Furthermore, while dermatophytes have been extensively studied, the mechanisms underlying keratin degradation by non-pathogenic fungi have not been explored to the same extent. One such fungus is *Onygena corvina*, a member of the Onygenales order, known for its ability to grow directly on keratinous substrates (20). Unlike dermatophytes, which invade host keratin, *O. corvina* is a saprophyte that thrives on decaying keratinous materials, suggesting it has evolved an efficient extracellular enzymatic system for keratin utilization (21). Previous studies have shown that culture supernatants of *O. corvina* can effectively degrade resilient keratin-rich materials, such as pig bristles (20). Another study, using duck feathers as substrate, also concluded that *O. corvina* is an efficient keratin degrader, far surpassing other fungi, such as *Trichoderma asperellum* (~23%) (21). Current data show that the broad-spectrum enzyme arsenal of *O. corvina* remains active across wide pH and temperature ranges, adding to the perception that *O. corvina* is a promising source for developing specialized keratinolytic enzyme blends (19–21).

In this study, we have employed a proteogenomic approach to gain comprehensive insight into the keratinolytic machinery of *O. corvina*. Our findings provide evidence of an intricate keratin degradation system that extends well beyond proteolysis and differs between wool and feathers. The identification of various cell wall-modifying enzymes, oxidoreductases, esterases, and phosphatases suggests hyphal penetration of the substrate and an important role for non-proteolytic enzymes that weaken the keratin structure. These results shed light on the complexity of microbial keratin degradation and show the need for a holistic approach to bioprocessing of keratin-rich biomass.

## RESULTS AND DISCUSSION

### Proteolytic potential of *O. corvina* based on genome and secretome data

For comprehensive proteomics studies, a high-quality reference database is essential (22). Since the publicly available genome for *O. corvina* was highly fragmented (GCA_000812245.1; 521 contigs), we used a combination of short reads (Illumina) and long reads (Oxford Nanopore Technology) sequencing to obtain a highly contiguous assembly (23). The new genome assembly (GCA_051362545.1) contains 13 contigs, yielding a genome size of 21.8 Mb and a completeness of 98.4%. Annotation of protein-coding genes was performed through an integrated approach that combined *de novo* predictions, predictions based on homology, and transcriptome-based (RNA-Seq) evidence. This workflow yielded 7,232 protein sequences, which were used to construct a custom database for subsequent proteomics studies.

**Fig 1:**
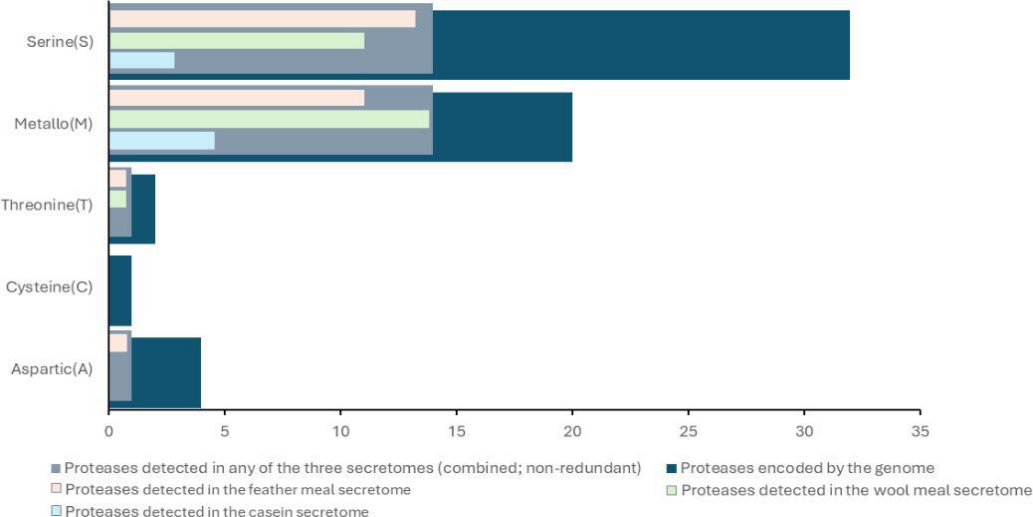
Putatively secreted proteases encoded by the *O. corvina* genome and those detected in the secretomes. The bar chart displays the distribution of proteases predicted to be secreted across five major MEROPS families: serine (S), metallo (M), threonine (T), cysteine (C), and aspartic (A). Dark blue bars represent all proteases predicted to be secreted, while grey bars indicate the count of unique proteases detected in the combined secretomes of *O. corvina* cultured on keratin-rich substrates (feather and wool meal) or casein (30 unique proteases in total). Light orange, green, and blue bars represent the number of proteases detected in the individual feather, wool, and casein secretomes, respectively (*n* = 26, 26, and 8 proteases in total, respectively).

Among these 7,232 proteins, 284 (3.9%) were predicted to be secreted based on the presence of a signal peptide and absence of transmembrane domains. A “Homology to Peptide Pattern” (Hotpep) search (24) identified 158 genes encoding proteases, whereas only 73 were predicted in the previous genome assembly (20). Of these 158 putative proteases, 80 contain a signal peptide for secretion, and of these, 59 lack a transmembrane domain and are thus predicted to be secreted. Table S1 presents an overview of all 80 signal peptide-containing putative proteases. The 59 secreted proteases are distributed across five major MEROPS (25) families: serine (S; *n* = 32), metallo (M; *n* = 20), cysteine (C; *n* = 1), threonine (T; *n* = 2), and aspartic (A; *n* = 4) (Fig. 1). Among these predicted secreted proteases, enzymes belonging to subfamilies S8, S9, S10, and M14, M28, M36 are the most abundant, with the largest number of proteases belonging to families S8 and M28 (*n* = 10 and 7, respectively). In the analysis of the secretomes of *O. corvina* during growth on casein, feather, or wool meal, which is described below, 30 of these 59 secreted proteases were detected in at least one of the secretomes (26 proteases for wool and feather meal, 8 for casein; in total 30 unique proteases; Fig. 1). The enrichment of S8 and M28 proteases in the genome is reflected in the secretome data: among the 30 secreted proteases detected, 8 belong to the S8 family and 5 to the M28 family.

### Secretome composition across the different substrates

To assess the ability of *O. corvina* to degrade various protein-rich sources, the fungus was grown on agar plates consisting of a minimal medium (see Materials and Methods) supplemented with feather meal, wool meal, or casein as the sole source of carbon and nitrogen. Casein was included instead of the keratin-rich substrates and represents an easily degradable reference protein source. Although *O. corvina* displayed similar radial growth on all three substrates, a clearance zone around the colonies was detected within 2 days of inoculation on casein, but only after 8 days on feather and wool meal (Fig. S1). This delayed detection of clearance zones during growth on recalcitrant keratin-rich substrates aligns with previous reports (26, 27).

To identify secreted proteins, samples were collected from the bottom agar of membrane plates containing one of the three substrates at 1, 2, and 3 days after inoculation. Of note, samples collected after day 3 were excluded from analysis due to the low number of detected proteins. High-resolution LC-MS/MS analysis, followed by protein quantification using the topN algorithm (28), showed high reproducibility, with most Pearson correlation coefficients being well above 0.8 and, in more than half of the cases, above 0.9 (Fig. S2 to S4). A total of 154 proteins detected in at least two of the three replicates for at least one of the three substrates (Table S2) were considered for further analysis. Among these, 80 proteins had a predicted signal peptide, of which 7 also contained transmembrane domains, yielding 73 putatively secreted proteins (Table S3; Fig. S5). On average, 52.7% of all identified proteins in the three secretomes were predicted to be secreted (Table S4). Given that only 3.9% of the total *O. corvina* proteome is putatively secreted, the membrane plate method effectively captured and significantly enriched for extracellular proteins.

**Fig 2:**
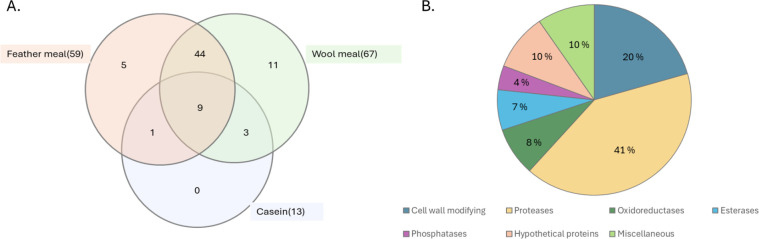
Number and functional annotation of 73 putatively secreted proteins detected in *O. corvina* secretomes. (**A**) Venn diagram representing the distribution of 73 putatively secreted proteins in the secretome during growth on feather meal, wool meal, or casein. The total number of detected proteins for each substrate is indicated in brackets. (**B**) Functional annotation of the 73 putatively secreted proteins. The figure depicts the proportion of different classes of proteins detected in the *O. corvina* secretome, irrespective of the substrates used for growth. The proteins were annotated using InterProScan, UniProt, and BLAST analysis, followed by manual curation and classification into the functional categories. Information regarding the nature and detection levels of these proteins can be found in Table S3; Fig. S5.

Focusing on the 73 putatively secreted proteins, the composition of the secretomes differed markedly between keratin-rich substrates and casein. The casein secretome included only 13 proteins, all of which were also present in the feather and wool secretomes, which contained 59 and 67 proteins, respectively (Fig. S5). The higher number of proteins secreted on keratin-rich substrates likely reflects the greater structural complexity of keratin, necessitating a more extensive enzymatic repertoire for degradation. Of the 60 proteins found exclusively in the keratin-grown secretomes, 44 were shared between the wool and feather meal secretomes (Fig. 2A), suggesting a common enzymatic machinery for the breakdown of both α- and β-keratin, alongside adaptations specific to each substrate.

Functional annotation revealed that proteases represent the largest category, making up 41% of the secretome (30 of 73 putatively secreted proteins), while other groups included cell wall-modifying enzymes, oxidoreductases, esterases, phosphatases, as well as hypothetical proteins and proteins with miscellaneous functions (Fig. 2B). Notably, hypothetical proteins with unknown functions accounted for 10% of the detected, putatively secreted proteins. The casein secretome was dominated by eight proteases alongside three hypothetical proteins, one esterase, and one oxidoreductase (Fig. S5). Overall, the presence of 60 putatively secreted proteins unique to the keratin-grown secretomes highlights their likely roles in keratin degradation.

### Secretion of proteases

Growth of *O. corvina* on keratin-rich substrates led to the secretion of a diverse range of proteases, primarily from the serine (S) and metalloprotease (M) families. Out of the 158 putative proteases encoded by the *O. corvina* genome, 35 were detected by proteomic analysis following growth on feather meal, wool meal, and/or casein. Of these, 32 proteases contained a predicted signal peptide, including two with accompanying transmembrane domains, yielding 30 putatively secreted proteases. The remaining three proteases, lacking a predicted signal peptide, were classified into the M1, M3, and M49 metalloprotease families.

**Fig 3:**
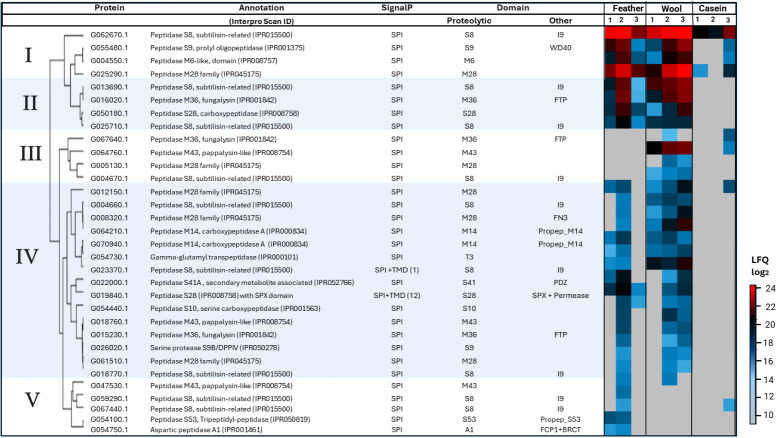
Heat map representation for 32 detected proteases with a standard signal peptide (SPI). Two of these proteases contain a transmembrane domain (TMD) and are thus not expected to be secreted. The figure shows Protein ID, SignalP 5.0, and TMHMM 2.0 predictions, the protease class, the presence of additional domains, and functional annotation of the protease according to InterProScan, and relative abundance (as log_2_ LFQ). Every row represents one protein, and the colors in the heatmap depict protein abundance (average of three replicates) during growth on minimal medium plates supplemented with 1% (wt/vol) feather meal, wool meal, or casein at day 1, 2, or 3. The scale of the heatmap ranges from high abundance (red color) to low abundance (light blue color). Gray color indicates that the protein was not detected. Proteases are divided into five hierarchical clusters based on protein abundance and expression pattern (Clusters I–V). Abbreviations: I9, Peptidase Inhibitor I9; WD40, 40 amino acid motif with terminal Trp-Asp (W-D) dipeptide; FTP, Fungalysin/Thermolysin propeptide; FN3, Fibronectin III domain; Propep_M14, M14 propeptide; PDZ, domain originally identified in PSD95, Dlg, and ZO-1 proteins; SPX, domain originally identified in SYG1, Pho81, and XPR1 proteins; Propep_S53, S53 propeptide; FCP1+BRCT, phosphatase and BRCA1 C-terminal domains.

A heat map of the 32 proteases with a predicted signal peptide revealed five distinct clusters (Fig. 3). Cluster I comprises high-abundance proteins detected with all three substrates. Of note, several of these proteases were more abundant and found at more time points for wool and feather meal, compared to casein. Cluster II contains high-abundance proteases detected exclusively during growth on the keratin-rich substrates. Clusters III, IV, and V comprise a wide variety of proteases with relatively low abundance. The proteases in Cluster IV were detected with both keratin-rich substrates, whereas Clusters III and V contain proteases unique to wool and feather meal, respectively.

Cluster I includes four highly abundant proteases that were detected with all substrates and, in the case of the keratin-rich substrates, across all time points. Two of these belong to the S8 and M6 families of *endo*-proteases, while the others are classified within the S9 and M28 families of *exo*-proteases. The presence of both *endo*- and *exo*-acting proteases suggests a synergistic mechanism for keratin degradation. Such synergism has been observed in an earlier study of *O. corvina* growing on pig bristles, in which a synergy between G062670.1 (corresponding to GenBank accession: KP290860.1; S8 protease) and G025290.1 (corresponding to GenBank accession: KP290838.1; M28 protease) was reported (20). The two *exo*-acting proteases in Cluster I belong to the S9 (G055480.1) and M28 (G025290.1) families, which are different and exhibit distinct substrate preferences. The S9 family includes proteases that cleave substrates from the N-terminus, typically preferring those with a proline in the penultimate position (25, 29). On the other hand, M28 aminopeptidases remove N-terminal residues with a preference for large hydrophobic amino acids like leucine (25). Interestingly, while M28 peptidases cannot accommodate proline in the penultimate position (30), S9 peptidases preferentially cleave when such a proline is present, which indicates that *O. corvina* secretes a complementary set of *exo*-proteases to maximize degradation efficiency. Since Cluster I proteases were also detected on casein, they may not be specifically induced by keratin but rather be proteases with general functions that are triggered by the mere presence of protein and peptides derived thereof. This aligns with a previous study showing that G062670.1 (corresponding to GenBank accession: KP290860.1), the most abundant protease detected in this study, is an enzyme with high cleavage efficiency and no apparent cleavage specificity (31).

Cluster II is enriched in *endo*-proteases from the S8 and M36 families, along with a carboxypeptidase from the S28 family, all of which were detected exclusively during growth on keratin-rich substrates. The involvement of M36 and S8 proteases in the degradation of native keratin has been well documented (11, 12, 32). The S28 family consists of carboxypeptidases that hydrolyze prolyl bonds, making these enzymes particularly relevant for the degradation of keratin, which is rich in proline (12, 33). The exclusive detection of Cluster II proteases on keratin-rich substrates suggests that they are keratin-specific; by targeting otherwise recalcitrant parts of the keratin, they may complement the broad-specificity proteases in Cluster I.

**Fig 4:**
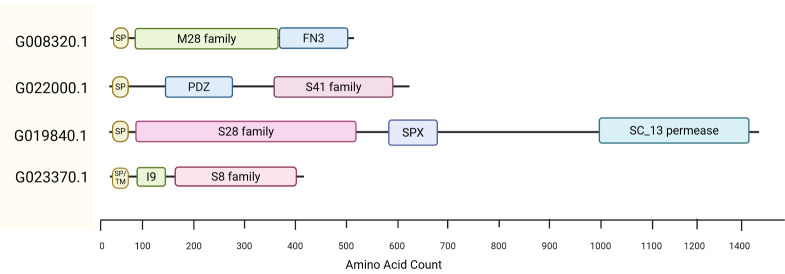
Domain architecture of multidomain proteases. Four multidomain proteases that were detected during growth on keratin-rich substrates and that are specifically discussed in the text are shown. The proteins are labeled with their accession number. The domains were assigned using InterProScan. The M28, S41, S28, and S8 domains belong to protease families, as classified in the MEROPS database. Abbreviations: I9, Peptidase Inhibitor I9; FN3, Fibronectin III domain; PDZ, domain originally identified in PSD95, Dlg, and ZO-1 proteins; SPX, domain originally identified in SYG1, Pho81, and XPR1 proteins; SC_13 permease, solute carrier 13 permease; SP, signal peptide; SP/TM, a signal peptide and 1 transmembrane helix.

Cluster IV, the cluster with the highest number of proteins, contains less abundant proteases detected exclusively during growth on keratin-rich substrates. It includes members from a diverse range of protease families, including the serine protease (S8, S9, S14), metalloprotease (M14, M28, M36, M43), and threonine protease (T3) families. A unique feature within this cluster is the presence of a C-terminal FN3 (fibronectin III) domain in G008320.1, a M28 family protease (Fig. 4). FN3 domains share topological similarity with immunoglobulin domains and exhibit diverse ligand-binding properties, potentially contributing to substrate specificity (34, 35). Another protease in this cluster, G022000.1, belonging to the S41 family, contains a PDZ domain (Fig. 4), which is a peptide-binding domain that may facilitate substrate binding (36, 37). Studies of a Vpr serine protease from *Bacillus cereus* DCUW showed that deletion of its C-terminal PDZ domain led to loss of feather degradation ability, while retaining activity against simpler substrates like casein and gelatin (38). These observations suggest that Cluster IV proteases may exhibit activity toward specific peptide sequences within keratin, acting in concert with Cluster II proteases to disrupt native keratin structures. Of note, based on previous studies on *T. rubrum* (39), the M14 carboxypeptidases (G064210.1*,* G070940.1) in Cluster IV likely require activation by subtilisin-like proteases from the S8 family.

Clusters III and V include proteases belonging to the S8, S53, A1, M36, M35, M43, and M28 families, some of which were only detected during the growth on wool meal (Cluster III) and some only during growth on feather meal (Cluster V). Notably, the detection of these proteins, on substrates enriched in α- or β-keratin, respectively, suggests that *O. corvina* tailors its proteolytic arsenal to accommodate distinctive biochemical properties of the substrates.

Interestingly, Cluster IV includes two proteases with transmembrane domains, an *endo*-protease from the S8 family (G023370.1) and an *exo*-protease from the S28 family (G019840.1). According to TMHMM 2.0 predictions, their catalytic domains are extracellularly oriented, suggesting a role in the breakdown of keratin-derived peptides before uptake by the fungal cell. Notably, the S28 family protease (G019840.1) also contains an SPX (SYG1/Pho81/XPR1) domain and a permease domain (Fig. 4). SPX domains (IPR004331) have been implicated in G-protein signal transduction and phosphate sensing (40). This domain structure suggests that G019840.1 may have multiple roles, including peptide processing, transport, and signal transduction.

**Fig 5:**
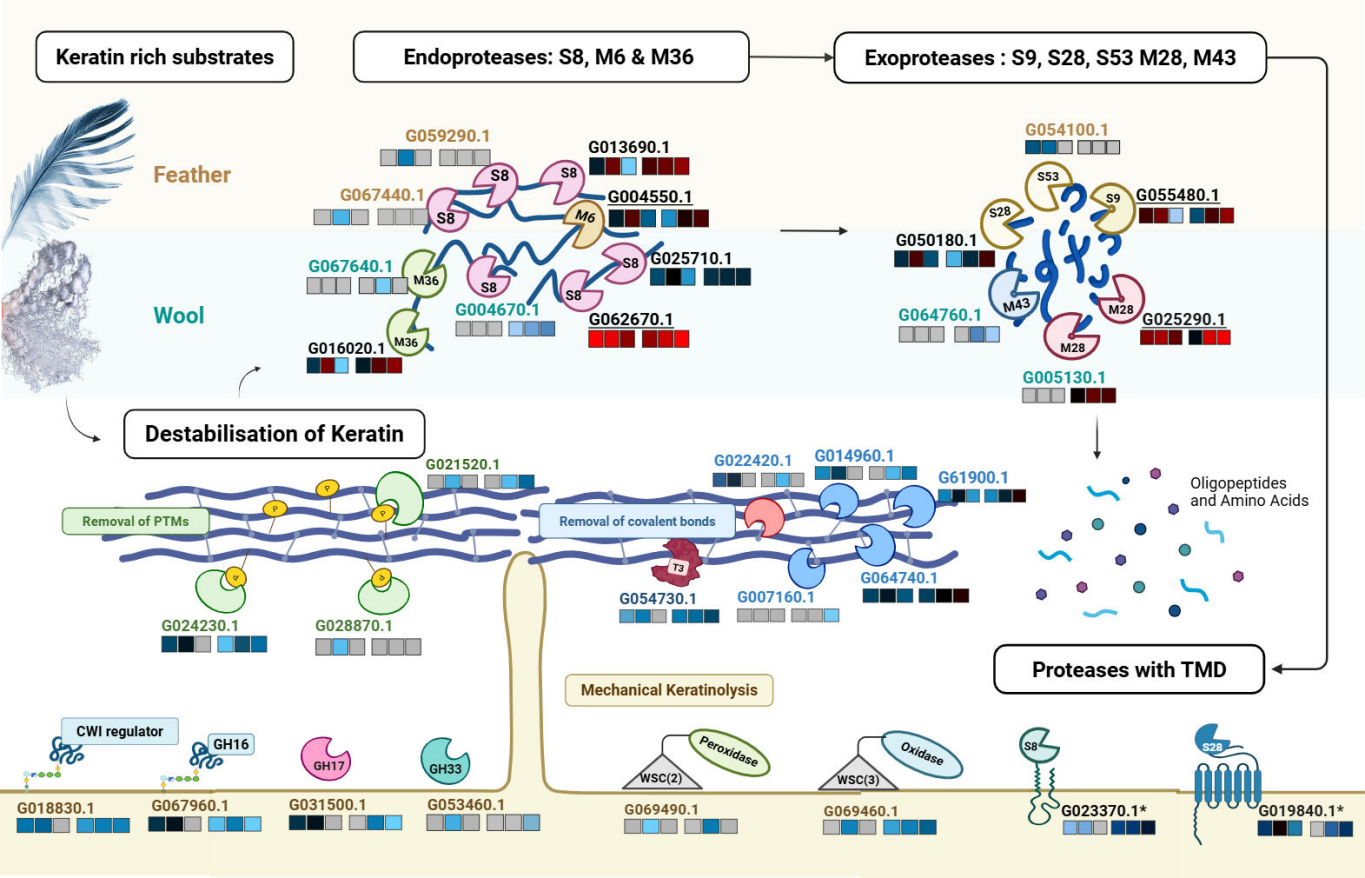
Overview of keratin degradation by *O. corvina*. Model for the degradation of keratin-rich substrates, such as feather and wool meal, by *O. corvina*, based on detected enzymes. Proteolysis involves the action of *endo*-proteases, *exo*-proteases, and proteases with TMD. Destabilization of keratin includes mechanical keratinolysis (cell wall-modifying enzymes), removal of post-translational modifications (PTMs) (phosphatases), and removal of covalent bonds (glutamyl transpeptidase, thioredoxin, and oxidoreductases). Heat maps above and below the proteins represent log_2_ LFQ intensities for proteins detected in feather (left) and/or wool (right) secretomes at days 1, 2, and 3. Table S3 lists the label-free quantification (LFQ) values for all the shown proteins. The figure shows proteases detected across all substrates (Cluster I, Fig. 3), those specific to keratin-rich substrates (Cluster II), those enriched on feather meal (Cluster V, except G054750.1 and G047530.1) or wool meal (Cluster III), and those with predicted TMDs. The MEROPS-based peptidase family classification is shown for each protease. Protein identifiers for proteases in Clusters I and II are shown in black (with underlining for Cluster I). Identifiers for proteins in Clusters III and V appear in blue and brown, respectively, while those with TMDs are marked with an asterisk (*). Detected enzymes likely involved in keratin destabilization include enzymes contributing to mechanical keratinolysis (G018830.1, G067960.1, G031500.1, G053460.1, G069490.1, G069460.1), phosphatases for removal of post-translational modifications (G021520.1, G024230.1, G028870.1), thioredoxin and oxidoreductases potentially involved in modifying disulfide bridges (G022420.1; G007160.1, G061900.1, G064740.1, G014960.1) and a T3 family gamma-glutamyl transpeptidase (G054730.1) involved in breaking iso-peptide bonds. The yellow “P” symbols indicate phosphate groups; CWI denotes cell wall integrity; WSC refers to mechanosensing domains characteristic of proteins involved in the CWI pathway. Note that, while this figure captures the information derived from the combined wool and feather secretomes, these two substrates are different and likely require slightly different enzyme machineries.

Altogether, the results show that *O. corvina* employs a comprehensive set of proteases in keratin degradation (Fig. 5). Cluster I proteases likely represent a general proteolytic machinery, hydrolyzing a broad range of proteins and peptides, while specialized proteases in other clusters target native keratin structures. The exclusive detection of Clusters III and V proteases in the wool and feather secretomes, respectively, shows that *O. corvina* tailors its enzymatic repertoire based on substrate composition and structure. By deploying a combination of both general and specific *endo*- and *exo*-proteases, possibly along with domain-specific adaptations that enhance substrate recognition and facilitate extracellular processing and peptide transport, the fungus ensures efficient keratin degradation, reinforcing its ability to thrive in keratin-rich environments.

### Expression of proteins involved in destabilizing keratin

Recent phylogenetic studies have positioned *O. corvina* within the dermatophytic *Arthrodermataceae* family (41, 42), suggesting that it may share keratin degradation strategies with dermatophytes. Given this classification, we speculated that the detection of proteins other than proteases (Fig. 2B) could be associated with mechanisms akin to adherence and invasion of skin keratin by dermatophytes (17). These additional proteins may play a role in destabilizing the keratin structure, thereby enhancing proteolytic access. Based on the functional diversity of the detected proteins (Fig. 2B), this destabilization appears to occur through three complementary mechanisms that are discussed below: (i) mechanical disruption of keratin fibrils, (ii) removal of post-translational modifications that reinforce keratin stability, and (iii) removal of disulfide and other covalent bonds that provide structural rigidity (Fig. 5).

#### Mechanical keratinolysis

Mechanical keratinolysis is a process in which certain fungi physically penetrate keratinized substrates using specialized structures like fronded mycelia or boring hyphae (43–45). These hyphae grow perpendicularly into the keratin matrix, facilitating colonization and subsequent enzymatic degradation. This phenomenon has been observed in fungi, such as *Microsporum gypseum* (46) and *T. rubrum* (27), but the underlying molecular mechanisms remain largely unknown.

*O. corvina* mycelia formed dense aggregates with feather or wool meal after 8 h of incubation in liquid minimal media. By 60 h, fragmented substrate was released from these aggregates (Fig. S6), suggesting keratin breakdown was likely mediated by mycelial adhesion. Notably, about 20% of the 73 putatively secreted proteins detected during growth on feather and wool meal are involved in cell wall assembly and remodeling (Fig. 2B). None of these were observed during growth on casein (Fig. S5), indicating a keratin-specific role. The fungal cell wall, primarily composed of glucans and chitin, is dynamic and undergoes extensive reorganization in response to environmental stimuli (47). The detected cell wall-active enzymes include glucosidases, transglycosylases, and sialidases that are known to be involved in cell wall synthesis and remodeling and that may facilitate the formation of mycelial structures that adhere to and mechanically disrupt keratin.

One such protein*,* G067960.1*,* is a GPI-lipid-anchored cell wall protein that is functionally annotated as a CRH1 transglycosylase of the glycosyl hydrolase (GH16) family (as defined in the CAZy database [48]). In *Saccharomyces cerevisiae*, CRH1 crosslinks β-1,3-glucan and β-1,6-glucan to chitin and localizes at sites of polarized growth, such as budding tips, reinforcing the cell wall under stress conditions (49, 50). Given its functional annotation and sequence similarity (51% sequence identity), the *O. corvina* CRH1 ortholog may have a comparable role, potentially strengthening the fungal cell wall during keratin penetration and degradation. Another protein, G031500.1, belongs to the GH17 family and is involved in β-1,3-glucan modification. It is similar to EglC from *Aspergillus* spp., which is an *endo*-1,3-β-glucosidase that is highly conserved in fungi and has a known role in cell wall remodeling associated with hyphal extension (51–53). Recent studies have indicated that members of the GH16 and GH17 families may function collaboratively as glucan and chitin transferases for cell wall biogenesis (54), supporting the hypothesis that cell-wall remodeling is important during growth of *O. corvina* on keratin. Sialidases (G053460.1; GH33 family) have been implicated in fungal cell wall stability, particularly under mechanical stress conditions. In *Aspergillus fumigatus*, deletion of a sialidase-encoding gene resulted in increased chitin deposition and unusual hyphal morphology, highlighting the role of this enzyme in maintaining cell wall integrity (55).

Additionally, the so-called cell wall integrity (CWI) pathway is critical for responding to mechanical stress (56). Proteins involved in this pathway are often equipped with so-called WSC domains acting as mechanosensors (57). Two of the detected secreted proteins (G069460.1*,* G069490.1) were found to contain such WSC sensor domains. Another detected protein, G018830.1, is homologous to ECM33, a GPI-anchored cell wall protein that is involved in the CWI pathway (58). The detection of these proteins suggests that *O. corvina* perceives keratin as mechanical stress, activating the CWI pathway in response.

The exclusive presence of proteins involved in cell wall synthesis and remodeling (GH16, GH17, and sialidases) along with cell wall integrity regulators (CWI pathway proteins) in the keratin secretomes, but not in the casein secretome, suggests involvement in mechanical keratinolysis.

#### Removal of post-translational modifications

Post-translational modifications (PTMs), such as phosphorylation, sumoylation, and glycosylation, play a crucial role in maintaining the structure and stability of keratin, the conformation of which is regulated by its PTM status (19, 59). The removal of PTMs may contribute to destabilizing keratin, and indeed, multiple enzymes putatively involved in removing PTMs were detected in the secretome of *O. corvina* growing on feather or wool meal (Fig. 5).

The phosphorylation of specific keratin-associated proteins has been implicated in the regulation of wool fiber crimping (60). These proteins form a matrix embedding keratin intermediate filaments that are cross-linked via disulfide bonds (61). We detected three phosphatases (G021520.1, G024230.1*,* G028870.1) annotated as alkaline phosphatase (IPR001952), histidine acid phosphatase (IPR016274), and calcineurin-like phosphoesterase (IPR014485) that could likely be involved in the dephosphorylation of these keratin-associated proteins. Additionally, detected enzymes, such as a β-hexoaminidase (G019890.1), a mannosidase (G049780.1), and two esterases (G036000.1*,* G042120.1), may be involved in modifying the glycosyl moieties found especially on the head domain of keratin polypeptide monomers. Such modifications will alter the nature of the filament head structures, ultimately leading to the disassembly of keratin filaments (13).

#### Removal of covalent bonds

Lastly, another key mechanism contributing to keratin destabilization is the removal of covalent bonds, such as disulfide bonds and keratin-specific isopeptide bonds (Fig. 5). The secretomes of keratin-grown *O. corvina* contained several enzymes potentially involved in these processes, including a γ-glutamyl transferase (G054730.1), a thioredoxin (G022420.1), and four distinct FAD-containing oxidoreductases (G007160.1*,* G061900.1*,* G064740.1*,* G014960.1). None of these proteins was detected during growth on casein.

Based on previous studies on keratin degradation by *Bacillus* sp. CH1, the γ-glutamyl transferase, is expected to cleave the isopeptide bonds between ε-amino groups of lysines and γ-glutamyl groups of glutamines, which commonly occur in keratinous proteins (62, 63). For disulfide bond reduction, the thioredoxin system has been widely studied. In this pathway, thioredoxins are maintained in their active redox state by FAD-containing thioredoxin reductases (64, 65). It is conceivable that the detected thioredoxin and FAD-containing oxidoreductases contribute to the reduction of disulfide bonds in keratin.

Taken together, the findings described so far suggest that keratin deconstruction in *O. corvina* is a multifaceted process integrating both mechanical and enzymatic processes (Fig. 5). The fungus appears to employ a largely conserved strategy across α- and β-keratin-rich substrates, leveraging a combination of specific and generalized responses. On the one hand, targeted secretion of enzymes, such as γ-glutamyl transferase, will lead to destabilization of the keratin, while on the other hand, broader cellular stress responses, such as activation of the CWI pathway, will contribute to fungal robustness during the interaction with keratin and, possibly, mechanical disruption. This intricate balance between specific enzymatic action and global stress adaptation likely enables *O. corvina* to efficiently degrade keratinous substrates in diverse environmental contexts.

### Substrate-specific keratinolytic response

As discussed above, *O. corvina* possesses a keratinolytic system capable of degrading both feather and wool substrates. The system contains many proteins that are used on both substrates, whereas each substrate specifically induces a small set of distinct secreted proteases. Feather meal stimulated production of proteases in Cluster V, whereas wool meal promoted production of proteases in Cluster III (Fig. 3). This substrate-specific induction is similar to regulatory mechanisms observed in lignocellulose-degrading filamentous fungi, in which production of specific cellulases is driven by substrate-derived inducers (66, 67).

**Fig 6:**
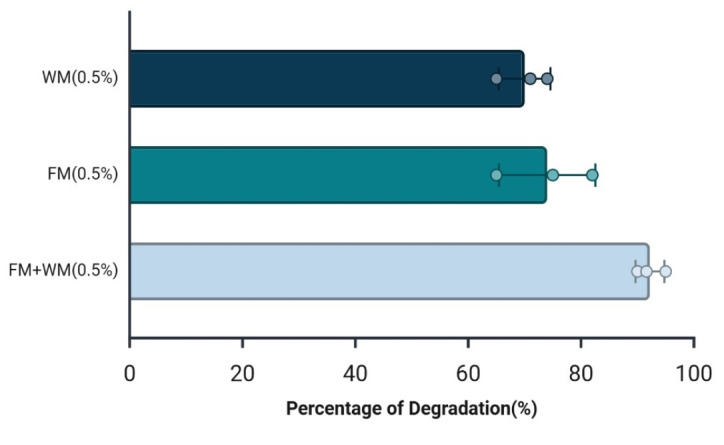
Degradation efficiency of feather meal, wool meal, and a combination thereof after 5 days of growth. Substrate degradation was measured by the weight loss method in cultures containing 0.5% (wt/vol) feather meal (FM), 0.5% (wt/vol) wool meal (WM), or 0.25% (wt/vol) of each of these two (FM+WM). The experiment was performed in triplicate, and all data points are shown.

To further investigate whether *O. corvina* modulates its keratinolytic response in a substrate-dependent manner, we assessed its substrate degradation efficiency when cultivated on individual versus combined keratin-rich substrates. Given the variation in proteolytic machineries induced by these substrates and the fact that *O. corvina* inhabits decaying environments where it may encounter both α- and β-keratin forms, we hypothesized that the combination of substrates would elicit a broader and more synergistic enzymatic response. Comparative analyses revealed that when cultivated on a combined feather and wool meal substrate, *O. corvina* achieved 95% degradation after five days of incubation at 25°C, a notable increase compared to approximately 70% degradation observed when grown on either feather or wool meal alone (Fig. 6).

While *O. corvina* biomass, as measured by dry weight, was comparable across the three culture conditions (Table S5), the specific proteolytic and keratinolytic activities of the supernatant were highest in the combined feather and wool cultures (Fig. S7 and S8), reaching values that were 1.2–1.4 times higher than those obtained with the individual substrates (Table S5). This increase in activity correlates with the observed enhancement in degradation efficiency and suggests that the proteolytic machineries induced by different keratin-rich substrates act synergistically.

### Concluding remarks

In this work, we have used a proteogenomic approach to gain insights into the synergistic mechanisms employed by *O. corvina* for degrading keratin-rich materials. We focused on β- and α-keratin-rich substrates, specifically feather and wool meal, and utilized agar plates to ensure enrichment of substrate-bound and secreted proteins in the secretomes. This latter method simulates the natural solid-state degradation of keratin substrates by keratinolytic organisms, enabling the detection of relevant proteins involved in the process, while minimizing (commonly observed) contamination with non-secreted proteins. Indeed, the secretomes discussed above were strongly enriched in proteins that are predicted to be secreted.

Our data show that *O. corvina* employs a complex keratin degradation mechanism. Next to having an extensive proteolytic machinery, the fungus likely destabilizes keratin through mechanical keratinolysis, removal of post-translational modifications, reduction of disulfide bonds, and cleavage of iso-peptide bonds, all of which enhance keratin accessibility for subsequent proteolysis. This process is facilitated by specific enzymatic activities (such as phosphatases and oxidoreductases) and a general stress response (including the CWI pathway). Proteolysis involves a combination of *endo*- and *exo*-proteases, several of which may have specific adaptations, in the form of additional domains, for substrate selectivity and peptide transport. As a consequence of these multiple mechanisms, keratin is converted into peptides and amino acids, making this recalcitrant material bioavailable for fungal uptake (Fig. 5). It should be noted that this degradation model is based on secretomes obtained during growth on substrates that had been subjected to pretreatment and that, consequently, the model may not capture the complexity of degrading the native forms of feathers and wool. Feathers and wool have quite different compositions, with wool being particularly complex, which likely means that different enzyme machineries are used for degrading these substrates. Indeed, our data shows that *O. corvina* to some extent adapts its enzyme repertoire to the nature of the keratinous substrate, suggesting that distinct keratin compositions provide complementary biochemical cues for the enzymatic response.

These novel insights into microbial degradation of keratin may guide the development of more efficient enzyme cocktails or microbial strains for keratin valorization. The complexity of the enzymatic machinery uncovered in this study indicates that the development of efficient enzyme cocktails is challenging, perhaps pointing to microbial conversion as the preferred way to go. In this respect, *O. corvina* and its keratinolytic system provide a promising starting point. Further studies of individual members of the diverse enzymatic repertoire of *O. corvina*, including proteases, oxidoreductases, esterases, phosphatases, and sialidases, are of interest, since these enzymes hold biotechnological potential. Ultimately, the present findings emphasize the need for a holistic approach in designing biotechnological solutions for processing keratin-rich byproducts, advancing keratin valorization, and promoting sustainable byproduct management.

## MATERIALS AND METHODS

### Strain and growth conditions

*O. corvina* (strain number: CBS 281.48; CBSKNAW Collection of the Westerdijk Fungal Biodiversity Institute, Utrecht, the Netherlands) was cultured on potato dextrose agar (PDA; Sigma-Aldrich, St. Louis, MO, USA) plates and subsequently cultivated on minimal medium plates supplemented with 1% (wt/vol) feather meal, wool meal, or casein. The composition of the minimal medium was as follows: 2 g/L magnesium sulfate heptahydrate (MgSO_4_ × 7H_2_O), 0.1 g/L potassium dihydrogen phosphate (KH_2_PO_4_), 0.01 g/L iron(II) sulfate heptahydrate (FeSO_4_ × 7H_2_O), 0.13 g/L calcium chloride dihydrate (CaCl_2_ × 2H_2_O), 10 g/L feather meal/wool meal/casein, and 15 g/L agar (VWR, Radnor, PA, USA). Casein (C7078) was purchased from Sigma-Aldrich (St. Louis, MO, USA). Feather and wool meal were prepared separately using raw materials provided by Norilia AS (Oslo, Norway). The materials were washed with warm running tap water until visually clean and then autoclaved at 130°C, 3 atm for 40 min. This material was subsequently dried at 100°C for 5 h (68). The dried materials were then ground using a bead beater with 50 mL stainless steel milling jars (Retsch, Haan, Germany) and 10 mm steel milling balls (VWR, Radnor, USA), for 5 min at 20 rpm (69). The resulting fine powder was stored in an airtight container at room temperature until further use.

For the preparation of agar plates for secretome analysis, membrane plates were prepared as described by Bengtsson et al. (70). Each plate consisted of two identical layers of the minimal medium supplemented with the respective substrate, which were separated by a sterile Supor 200 membrane (0.2 µm pore size, 47 mm diameter; Pall Life Sciences, Port Washington, USA). This membrane facilitates the separation of fungal cells, which stay in the upper agar layer, from the secreted enzymes that diffuse through the filter into the lower agar layer.

Plates containing the respective substrates and without a membrane were inoculated by placing a 4 mm agar plug from potato dextrose agar plates at the center of the plate. After incubation at 25°C for 2 weeks, agar plugs from these plates were transferred to membrane-containing minimal agar plates containing the corresponding substrate. The plates were then incubated at 25°C for 1, 2, and 3 days, after which samples for secretome analysis were collected.

### Sample preparation for proteomics

At each time point, agar discs from nine independent membrane plates (three biological replicates for each of the three substrates) were collected by punching out discs with an approximate volume of 100 µL from the bottom agar layer (70). Each disc was mixed with an equal volume of extraction buffer (10% SDS, 20 mM DTT, 100 mM Tris-HCl, pH 7.9) and incubated at 95°C for 10 min to dissolve the agar and solubilize all proteins, also those bound to substrate. After vortexing and centrifugation (10 min at 5,000 × *g*), the supernatant was concentrated to 10–15 µL using a vacuum centrifuge (SpeedVac, Thermo Fisher Scientific, Waltham, USA) and loaded on an SDS-PAGE gel, which was run at 220 mV for 2 min. Protein bands were stained with Coomassie brilliant blue, destained overnight, and excised. The gel pieces were washed, decolorized twice with 200 µL of 50% acetonitrile/25 mM ammonium bicarbonate, dehydrated by washing with 100 µL of 100% acetonitrile, and air-dried before reduction (50 µL of 10 mM DTT, 100 mM ammonium bicarbonate at 56°C for 30 min) and alkylation (50 µL of 55 mM iodoacetamide, 100 mM ammonium bicarbonate at room temperature in the dark for 30 min). Following removal of the excess alkylation solution, 200 µL of 100% acetonitrile was added to the gel pieces and incubated for 15 min at room temperature. After air drying, proteins in the gel pieces were digested overnight at 37°C with 30 µL of 10 ng/µL trypsin solution (Promega, Mannheim, Germany) (71). Digestion was stopped by adding 30 µL TFA to a final concentration of 0.5% (vol/vol). After sonication for 15 min, the peptides were desalted using a STAGE-TIP protocol (72), dried under vacuum, and dissolved in 10 µL of 0.1% (vol/vol) formic acid.

### Proteomic analysis

The peptide samples were analyzed using a nano-UPLC (nanoElute 2, Bruker) coupled to a trapped ion mobility spectrometry/quadrupole time-of-flight mass spectrometer (timsTOF Pro, Bruker). Peptide separation was performed on an Aurora C18 reverse-phase analytical column (1.6 µm, 120 Å, 25 cm × 75 µm) with an integrated emitter (IonOpticks, Melbourne, Australia), maintained at 50°C using an integrated oven. The column was equilibrated at 800 bar before sample loading. Peptides were eluted at a flow rate of 300 nL/min using a solvent gradient from 5% to 25% solvent B over 40 min, followed by an increase to 37% over 5 min. The solvent composition was then raised to 95% solvent B over 5 min and maintained for an additional 10 min, resulting in a total run time of 60 min. Solvent A consisted of 0.1% (vol/vol) formic acid in Milli-Q water, while solvent B was 0.1% (vol/vol) formic acid in LC-MS grade acetonitrile. The timsTOF Pro was operated in positive ion data-dependent acquisition PASEF mode and controlled using Compass Hystar version 6.2.1.13 and timsControl version 5.0.4. The acquisition mass range was set to 100–1,700 m/z, with TIMS settings: 1/K0 Start 0.85 V⋅s/cm² and 1/K0 End 1.4 V⋅s/cm², ramp time 100 ms, ramp rate 9.42 Hz, duty cycle 100%. The capillary voltage was set to 1,400 V, dry gas at 3.0 L/min, and dry temperature at 180°C. The MS/MS settings included 10 PASEF ramps, a total cycle time of 0.53 s, a charge range of 0–5, a scheduling target intensity of 20,000, an intensity threshold of 2,500, active exclusion release after 0.4 min, and CID collision energy ranging from 27 to 45 eV.

Protein quantification was performed using the MSFragger v4.0 search engine (28, 73) within FragPipe v20.0, employing the label-free quantification (LFQ)-match-between-runs (MBR) workflow. A closed search (74) was conducted against the predicted *O. corvina* proteome (PRJNA1280007; 7,232 proteins) supplemented with common contaminants and reversed decoy sequences for estimation of the false discovery rate (FDR). LFQ with MBR was performed using IonQuant (75) v1.9.8, allowing the determination of relative protein abundance between samples expressed as LFQ intensities. Peptide-spectrum matches (PSMs) were validated using Percolator (76), and protein inference was carried out with ProteinProphet (77). FDR filtration thresholds were set at 1% at both PSM and protein levels using Philosopher v5.0.0, employing a standard target-decoy database approach (78). Carbamidomethylation of cysteines was set as a fixed modification. Variable modifications included methionine oxidation, N-terminal acetylation, and pyro-glutamic acid formation at N-terminal glutamines. One missed cleavage was allowed. Normalization was applied in IonQuant to correct for technical variability and ensure comparability of protein abundance values across runs (74). The output was further processed in Perseus (79) v1.6.15.0. Proteins identified as potential contaminants, reverse hits, or proteins only identified by a single peptide were excluded. Proteins were considered detected only if identified in at least two of three replicates. The LFQ intensities were log_2_-transformed prior to analysis. Hierarchical clustering and heatmap generation were done with Euclidean distance measure and complete linkage. Functional annotation of the detected proteins was then refined using InterProScan (80) version 5.73-104.0 and the MEROPS database (25). The subcellular localization of the detected proteins was predicted using a combination of SignalP 5.0 (81) and TMHMM (82) v2.0.

### Substrate degradation and enzyme activity assays

To assess substrate degradation dynamics, *O. corvina* was cultured in 300 mL Erlenmeyer flasks containing 100 mL minimal medium (composition described above) and 0.5 g of feather meal, wool meal, or a combination of feather and wool meal (each at 0.25 g). The flasks were inoculated with 1 g of wet mycelium. All flasks were incubated on a rotary shaker at 100 rpm and 25°C for up to 5 days. The experiment was performed in triplicate. Substrate degradation was monitored visually and recorded through images taken from the bottom of the conical flasks at 0, 8, and 60 h. After 5 days of incubation, substrate degradation was evaluated using the weight loss method (83). The insoluble material was collected onto a funnel lined with miracloth (Merck KGaA, Darmstadt, Germany). The mycelial biomass was removed with forceps and then washed first with 10% sodium hydroxide (84) at 25°C for 5 min, until most of the adhering substrate was removed, and then with water. The washing liquid was returned to the funnel with miracloth, ensuring that all remaining substrate was collected. The washed mycelial biomass was dried at 65°C for 24 h, and the dry weight was recorded. The amount of residual substrate was determined by placing the miracloth (we pre-recorded the weight both before and after the filtration) in a moisture analyzer (SARTORIUS MA160 Moisture Analyzer, Sartorius AG, Göttingen, Germany), for the determination of the moisture content. The final dry weight of the remaining substrate was calculated by subtracting the moisture weight from the wet weight of the residual substrate. The percentage of degradation was calculated using the formula:

Percentage of Degradation (%) = [(0.5 – Dry weight of residual substrate)/0.5] × 100.

To assess whether the short washing step with NaOH causes artifacts due to chemical degradation of the keratin substrate, control experiments were performed in which the washing step was performed using phosphate-buffered saline (PBS; 100 mL, 25°C, 15 min). Both washing procedures yielded similar results, both in terms of the degree of substrate degradation and the amount of mycelial biomass (Fig. S9), showing that artifacts due to the use of NaOH are unlikely.

Supernatants from the cultures with feather meal, wool meal, or the combined substrate were diluted in 50  mM potassium phosphate buffer (pH 6.0) to normalize protein concentration across all samples. These protein-normalized supernatants were filtered through 0.2 µM filters (Merck KGaA, Darmstadt, Germany) and used to assess proteolytic and keratinolytic activities. Each reaction (200 µL total volume) contained 100 µL of the diluted supernatant and 100 µL of either a 2% (wt/vol) azocasein solution (for proteolytic activity; Megazyme, Bray, Ireland) or a suspension containing 2% (wt/vol) keratin azure (for keratinolytic activity; Sigma-Aldrich, St. Louis, USA), both prepared in the same buffer. Control reactions containing either substrate or supernatant alone were included and did not show significant background signals. The volume of undiluted supernatant was limited to 1%–2% of the total reaction volume to maintain assay stability.

Reactions were set up under aseptic conditions and incubated at 37°C at 1,000 rpm for 30 to 120 min for proteolytic activity or 8 to 24 h for keratinolytic activity. For azocasein-containing samples, the reaction was terminated by adding 600 µL of 5% trichloroacetic acid (TCA; Sigma-Aldrich, St. Louis, MO, USA), followed by centrifugation at 5,000 × *g* for 10 min. Reactions containing keratin azure were also centrifuged under the same conditions. The resulting supernatants were collected and transferred to a 96-well plate. Absorbance was measured at 440 nm for proteolytic activity and at 595 nm for keratinolytic activity using a multimode microplate reader (Varioskan LUX, Thermo Fisher Scientific, Waltham, USA). Based on values typically used in the literature, one keratinase unit (U) was defined as the amount of enzyme that produces an increase of 0.01 in absorbance at 595 nm per hour, whereas one protease unit was defined as the amount of enzyme that produces an increase of 0.01 in absorbance at 440 nm per hour.

## Data Availability

The mass spectrometry data have been deposited to the ProteomeXchange Consortium via the PRIDE (85) partner repository with the data set identifier PXD065899. The genome has been made available in the NCBI GenBank under the accession number PRJNA1280007. The gene and protein annotations, putative functional annotation, and CAZyme predictions are available on Figshare (https://doi.org/10.6084/m9.figshare.29328893.v1).

## SUPPLEMENTAL MATERIAL

Supplemental Material aem.01727-25-s0001.docx Figures S1 to S9; Tables S4 and S5.

Table S1 aem.01727-25-s0002.xlsx List of 80 genome-predicted proteases of *O. corvina* with a signal peptide and their detection in the feather meal, wool meal, and casein secretomes.

Table S2 aem.01727-25-s0003.xlsx Log_2_ LFQ intensities and predicted signal peptide and transmembrane domains for all 154 detected proteins.

Table S3 aem.01727-25-s0004.xlsx Log_2_ LFQ intensities for 80 detected proteins with a predicted signal peptide.

ASM does not own the copyrights to Supplemental Material that may be linked to, or accessed through, an article. The authors have granted ASM a non-exclusive, world-wide license to publish the Supplemental Material files. Please contact the corresponding author directly for reuse.
