# Supplementary material for: Proteolytic and non-proteolytic mechanisms of keratin degradation in Onygena corvina revealed by a proteogenomic approach
Source: Appl Environ Microbiol. 2025 Nov 28;91(12):e01727-25. doi: 10.1128/aem.01727-25 (PMC12724343; doi:10.1128/aem.01727-25)
Supplement: Supplemental Material — Figures S1 to S9; Tables S4 and S5. [file aem.01727-25-s0001.docx]

**Proteolytic and Non-Proteolytic Mechanisms of Keratin Degradation in *Onygena corvina* Revealed by a Proteogenomic Approach**

Siddhi Pavale^1#^, Clémentine Isembart^2^, Volha Shapaval^2^, Tina R Tuveng^1^, Sabina Leanti La Rosa^1^, Vincent G.H. Eijsink^1^

^1^Faculty of Chemistry, Biotechnology and Food Science, Norwegian University of Life Sciences (NMBU), 1433 Ås, Norway

^2^Faculty of Science and Technology, Norwegian University of Life Sciences (NMBU), 1433 Ås, Norway

#Address correspondence to Siddhi Pavale, [siddhi.pavale@nmbu.no](mailto:siddhi.pavale@nmbu.no)

**Supplemental Material**


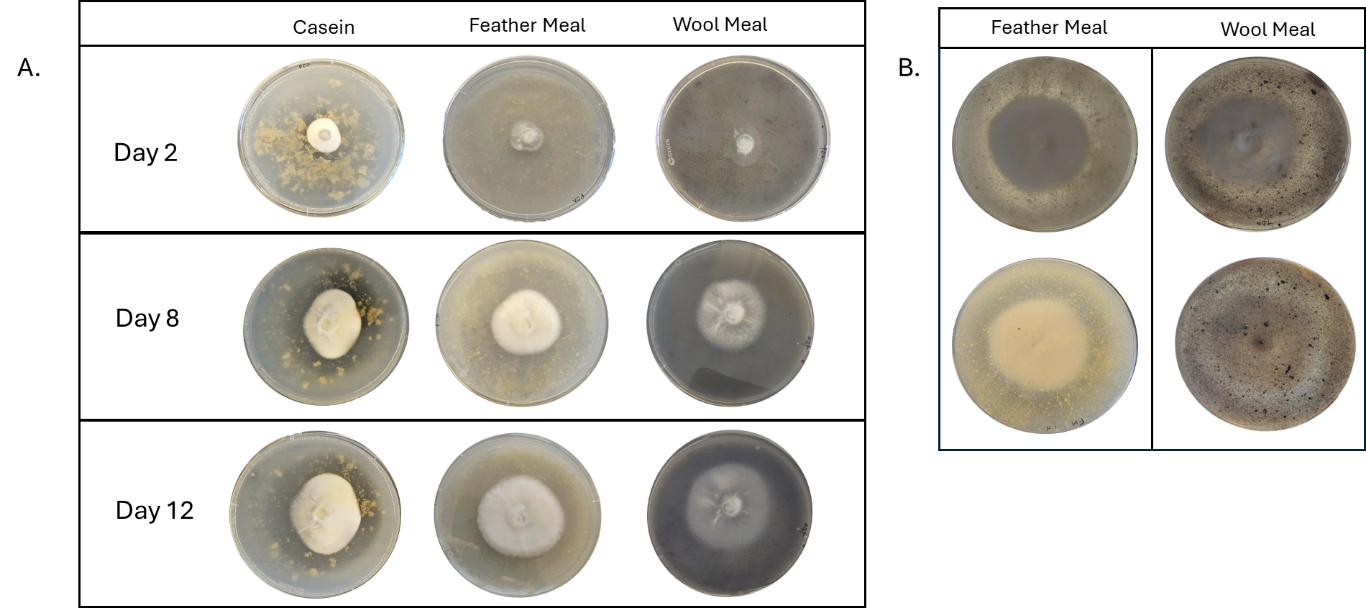


**Figure S1:** **Growth and clearance zone of *O. corvina* growing on 1% casein, feather meal, or wool meal.** (A) Radial growth of fungal colonies on minimal medium plates containing 1% casein, feather meal, or wool meal as the sole carbon and nitrogen source. Images were taken on days 2, 8, and 12 after incubation at 25 °C. A zone of clearance is visible around the colony on the casein plates. Due to the relatively low solubility of the keratin-rich substrates, clearance zones are less distinct for feather and wool meal. (B) To improve the visibility of clearance zones on feather and wool meal, the top (upper row) and bottom (lower row) views of plates at day 12 were photographed against bright light.

*
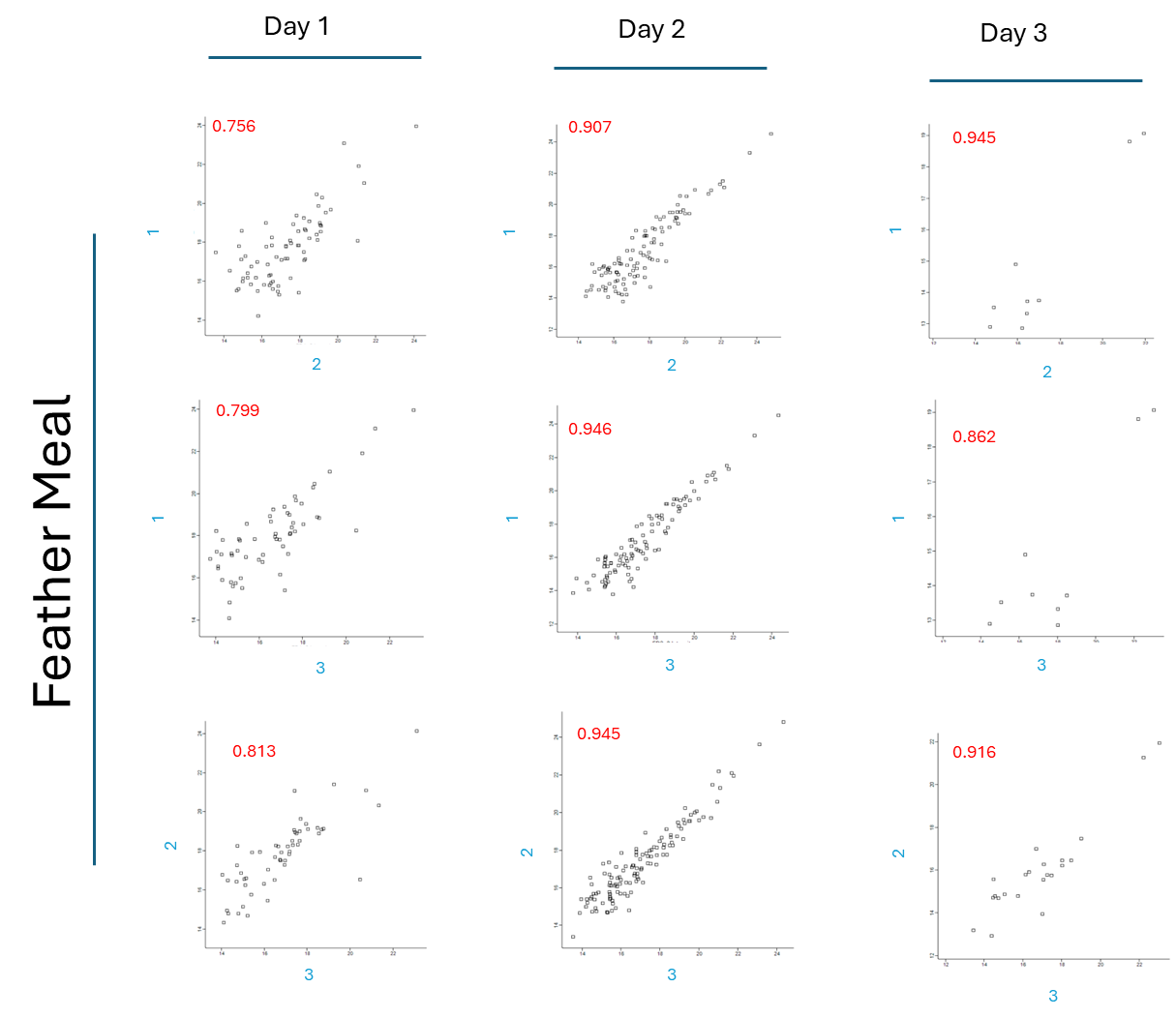
*

**Figure S2: Multi-scatter plots showing pairwise comparisons of biological replicates for the feather secretome at Day 1, Day 2, and Day 3.** Each scatter plot indicates the protein abundance values (log_2_ LFQ intensity) of two replicates at a given time point (Replicates 1–3), plotted on the x- and y-axes. Each dot represents a protein detected in both replicates. The Pearson correlation coefficient (r) between each replicate pair is displayed in red within each plot.


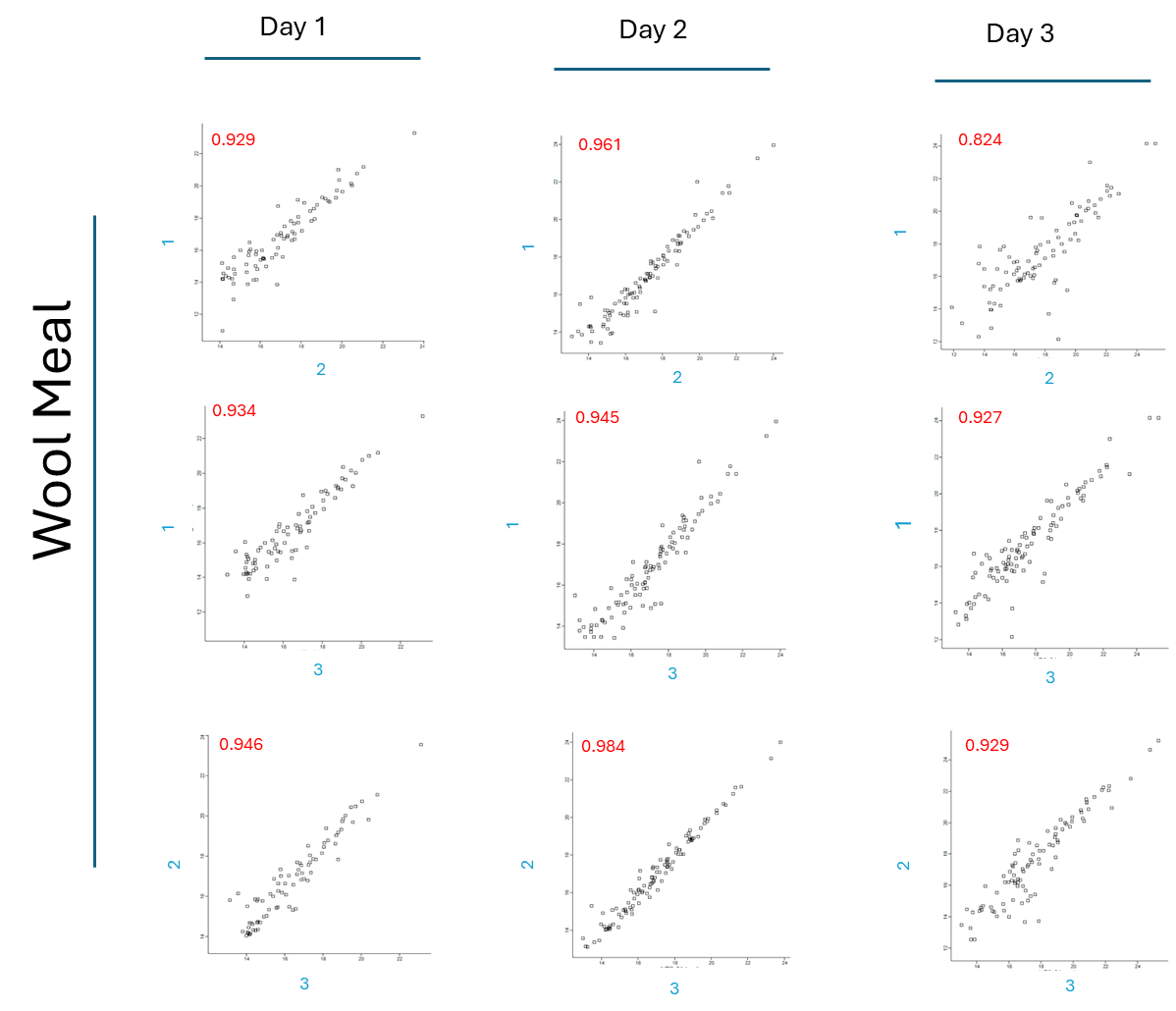


**Figure S3: Multi-scatter plots showing pairwise comparisons of biological replicates for the wool secretome at Day 1, Day 2, and Day 3.** Each scatter plot indicates the protein abundance values (log_2_ LFQ intensity) of two replicates at a given time point (Replicates 1–3), plotted on the x- and y-axes. Each dot represents a protein detected in both replicates. The Pearson correlation coefficient (r) between each replicate pair is displayed in red within each plot.


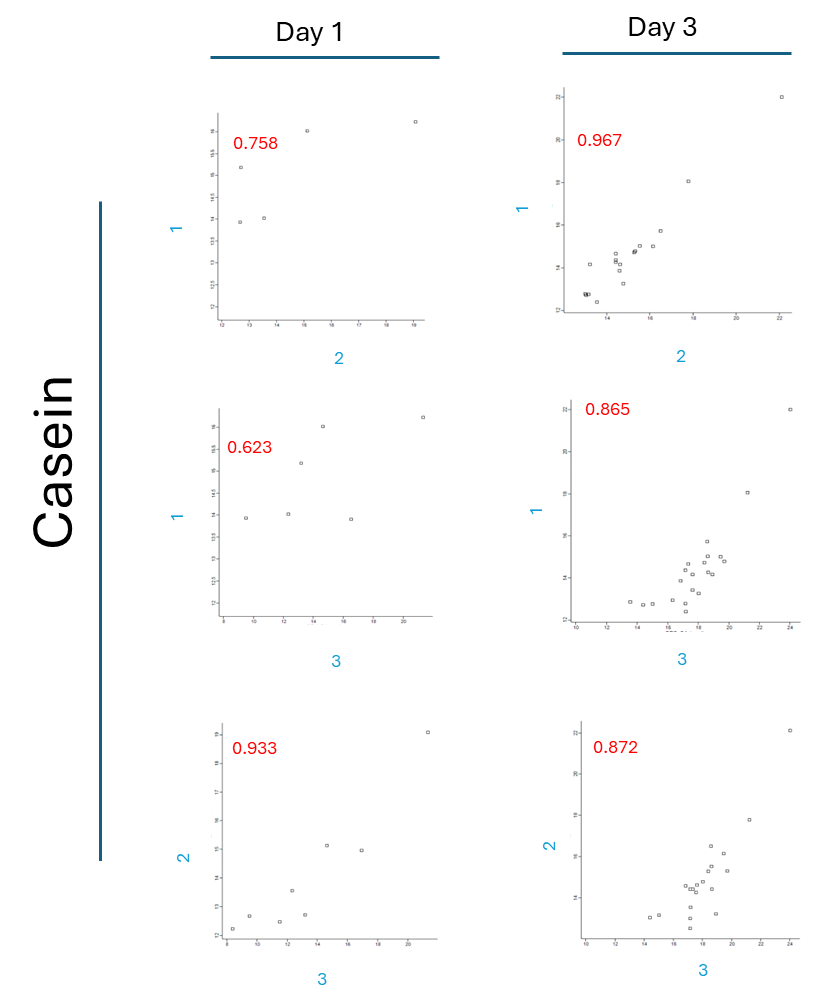


**Figure S4: Multi-scatter plots showing pairwise comparisons of biological replicates for the casein secretome at Day 1, Day 2, and Day 3.** Each scatter plot indicates the protein abundance values (log_2_ LFQ intensity) of two replicates at a given time point (Replicates 1–3), plotted on the x- and y-axes. Each dot represents a protein detected in both replicates. Since only one protein (a protease) was detected on day 2 with casein, this timepoint is not included in this analysis. The Pearson correlation coefficient (r) between each replicate pair is displayed in red within each plot.

***
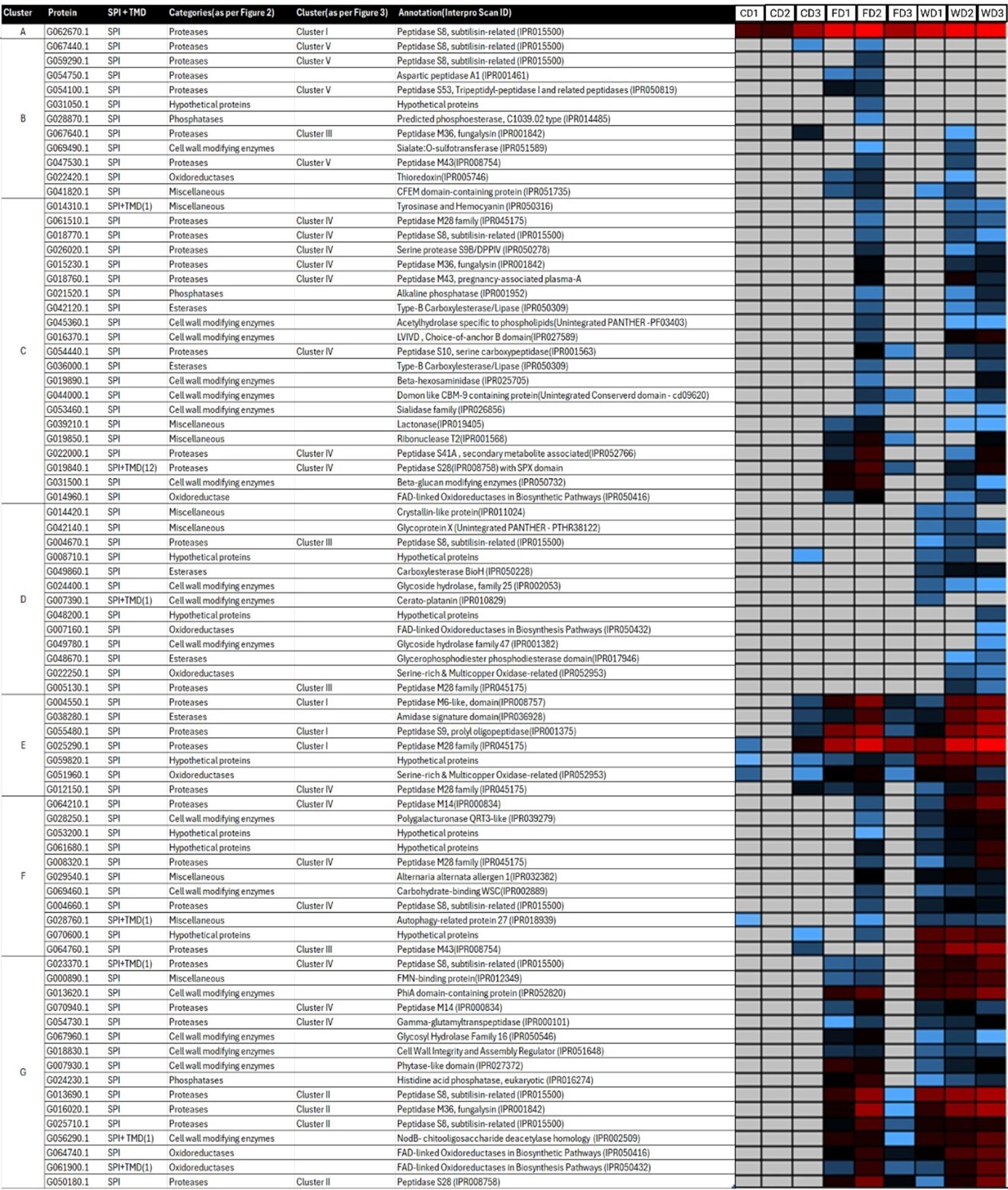
*Figure S5: Functional annotation and heat map representation of 80 proteins (with predicted signal peptide – SPI) during growth on feather meal (FD1, FD2, FD3), wool meal (WD1, WD2, WD3), or casein (CD1, CD2, CD3) at day 1, 2, and 3**. Each row represents a protein labeled with its accession number, and the color intensity reflects protein abundance (average of three replicates) at days 1, 2, or 3 on feather meal, wool meal, or casein. Note that seven of these proteins contain one or more transmembrane domains (indicated by “TMD”) and are thus not likely to be secreted. The heat map scale ranges from high abundance (red) to low abundance (light blue). Grey indicates proteins not detected. Proteins are divided into hierarchical clusters based on protein abundance (A, B, C, D, E, F, and G). The table also shows functional categories (as per Figure 2B), protease clusters (I-V; as per Figure 3), and annotations according to InterProScan (entry name and accession number, representing a protein superfamily, family, domain or repeat). In some cases, for entries lacking in InterPro, the PANTHER/CDD accession number is provided.


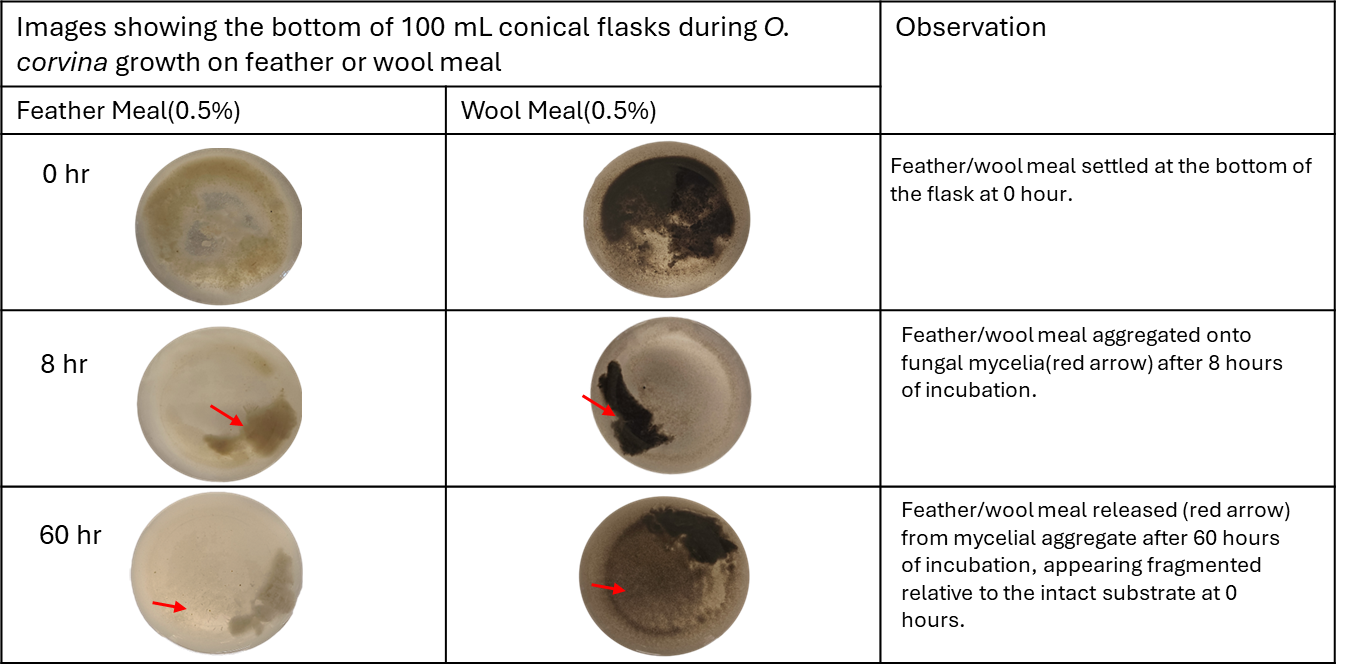


**Figure S6: Live cell-substrate contact during growth of *O. corvina* on feather or wool meal**. Images show the bottom of 100 mL conical flasks containing *O. corvina* cultures inoculated with 1 g of freshly cultivated mycelium in minimal media supplemented with 0.5% (w/v) feather meal or wool meal as the sole source of carbon and nitrogen. Cultures were incubated at 25 °C with shaking at 100 rpm, and images were captured at 0, 8, and 60 hours of incubation. The release of substrate from the mycelial aggregate at 60 hours is more clearly visible for wool meal than feather meal, because of better contrast against the background.

**Figure S7. Proteolytic and keratinolytic activities in *O. corvina* cultures grown on feather meal, wool meal, and a combined substrate.** Supernatants from *O. corvina* cultures grown for 5 days in feather meal (0.5%), wool meal (0.5%), or a combination of both (0.25% each) were tested for proteolytic and keratinolytic activities. Reactions were performed in 50 mM potassium phosphate buffer (pH 6.0) at 37 °C and supernatants were dosed such that all reactions contained the same amount of protein. Proteolytic activity was assessed using 1% azocasein, with absorbance measured at 440 nm after 120 minutes. Keratinolytic activity was measured using keratin azure (1% w/v), with absorbance recorded at 595 nm after 24 hours. Fig. S8 shows progress curves for degradation of keratin azure over time that were recorded in an independent experiment and that shows the same type of activity differences between the culture supernatants.

**Figure S8. Increase in absorbance at 595 nm over time, reflecting the keratinolytic activity of *O. corvina* culture supernatants.** Protein-normalized supernatants from *O. corvina* cultures grown on feather meal (0.5%), wool meal (0.5%), or a combined substrate (0.25% of each) were incubated with 1% keratin azure at 37 °C, and absorbance at 595 nm was measured after 8, 16, and 24 hours. Data represent mean values of triplicates. Specific activities are reported in Table S5.


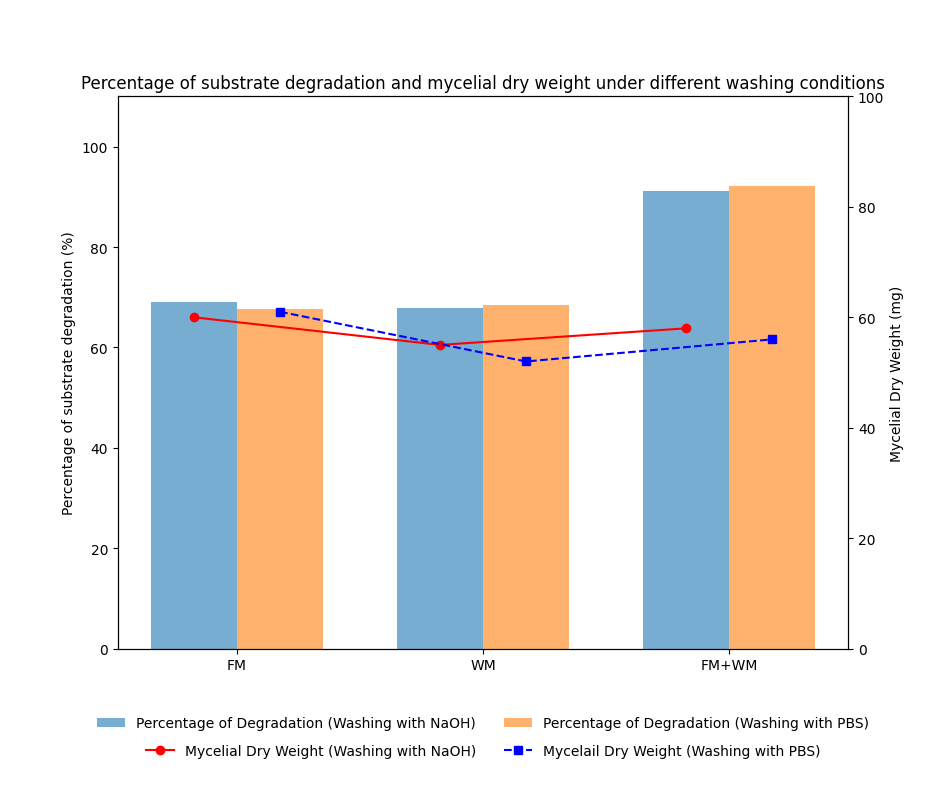


**Figure S9. Percentage of substrate degradation and mycelial dry weight for *O. corvina* grown on different substrates and using different washing conditions in the analysis.** Cultures were grown for 5 days on feather meal (0.5%), wool meal (0.5%) or a combination of both (0.25% of each). The amounts of mycelial biomass and the degree of substrate degradation were then determined as described in the Materials and Methods section, using either 10 % NaOH (blue bars and red data points) or PBS (orange bars and blue data points) in the washing step.

**Table S1, S2 and S3 have been uploaded separately under ‘Supplemental Material’ due to their large size.**

**Table S1: List of 80 genome-predicted proteases of *O. corvina* with a signal peptide and their detection in the feather meal, wool meal and casein secretomes.** The table lists 80 proteases encoded by the *O. corvina* genome that contain an SPI signal peptide. For each protein, the accession number, MEROPS family annotation (as per Hotpep search), presence of a predicted signal peptide (SPI) and a transmembrane domain (TMD), and detection in the secretome during growth on feather meal (FM), wool meal (WM), or casein are provided. Detection is indicated by “yes.” MEROPS family annotations are specified for serine (S), metalloproteases (M), aspartic (A), threonine (T), and cysteine (C) proteases.

**Table S2: Log₂ LFQ intensities and predicted signal peptide and transmembrane domains for all 154 detected proteins.** The Table contains the Log₂ LFQ intensities at day 1, 2, and 3 of cultivation for casein (CD1, CD2, CD3), feather meal (FD1, FD2, FD3), and wool meal (WD1, WD2, WD3). Each row contains the accession, the signal peptide (SPI) and transmembrane domain (TMD) prediction status and Log₂ LFQ intensities for each substrate at different timepoints (average of triplicates).

**Table S3: Log₂ LFQ intensities for 80 detected proteins with a predicted signal peptide.** The table contains the Log₂ LFQ intensities at day 1, 2, and 3 for casein (CD1, CD2, CD3), feather meal (FD1, FD2, FD3), and wool meal (WD1, WD2, WD3) secretomes. Each row contains the accession, the signal peptide (SPI) and transmembrane domain (TMD) prediction status, the assigned functional category (as per Figure 2B), a functional annotation based on InterProScan, and the Log₂ LFQ values. The InterProScan annotation includes the entry name and accession number, representing a protein superfamily, family, domain or repeat. In some cases, for entries lacking in InterPro, the PANTHER/CDD accession number is provided.

| **Source** | **Total number of detected proteins** | **Total number of putatively secreted proteins** | **Percentage of secreted proteins (%)** |  |  |
| --- | --- | --- | --- | --- | --- |
| Casein secretome (control) | 24 | 13 | 54 | 52.7 | Percentage of putatively secreted proteins across all three substrates (average) |
| Feather meal secretome | 118 | 59 | 50 |  |  |
| Wool meal secretome | 125 | 67 | 54 |  |  |
| Genome-predicted proteome | 7233 | 284 | 3.9 |  |  |

**Table S4:** **Percentage of secreted proteins in the *O. corvina* genome and in the various substrate-specific secretomes.** The table shows the total number of detected proteins, the number of putatively secreted proteins, and the corresponding percentage of secreted proteins for each of the casein, feather and wool secretomes. It also includes the average percentage of putatively secreted proteins across the three substrate-specific secretomes. For comparison, similar numbers are provided for the predicted proteome of *O.corvina*.

**Table S5: Fungal biomass and enzyme activity in *O.corvina* cultures grown on feather meal, wool meal or a combination of both substrates.** The table Fungal biomass production (as dry weight), specific keratinase and proteolytic activity of *O.corvina* cultures grown on feather meal (0.5%), wool meal (0.5%), and a combination of both substrates (0.25% of each).

| **Conditions** | **Dry weight of fungal biomass (mg) ± SD** | **Specific Keratinase Activity (U/mg) ± SD** | **Specific Proteolytic Activity (kU/mg) ± SD** |
| --- | --- | --- | --- |
|  |  |  |  |
| Feather meal (0.5%) | 63 ± 2.6 | 164 ± 11 | 2.98 ± 0.17 |
| Wool meal (0.5%) | 55 ± 2 | 195 ± 5 | 2.82 ± 0.07 |
| Feather + Wool meal (0.25%) | 59 ± 3.1 | 241 ± 9 | 3.53 ± 0.11 |
